# Formulation, characterization and evaluation of cytotoxic potential for febuxostat loaded chitosomes against cervical cancer cells (Hela cells)

**DOI:** 10.1038/s41598-026-58557-0

**Published:** 2026-06-21

**Authors:** Wedad Sakran, Mai Abdel‑Hakim, Rania S. Abdel‑Rashid

**Affiliations:** Pharmaceutics and Industrial Pharmacy Department, Faculty of Pharmacy, Capital University, Ain Helwan, Cairo, 11795 Egypt

**Keywords:** Chitosomes, Repurposing, Cervical cancer, Febuxostat, Hela cells, Biotechnology, Cancer, Drug discovery, Nanoscience and technology

## Abstract

The presented study is focused on evaluating the efficiency of repurposing Febuxostat (FBX) as a chemotherapeutic newcomer against the cervical cancer cell line (Hela cells). Exploiting nanotechnology benefits in drug delivery, FBX was incorporated into chitosan-coated niosomes (Chitosomes). Using a 2^3^ factorial design, eight formulations were characterized for their entrapment efficiency percentage, particle size, and zeta potential. The optimum (FBX) loaded chitosomes displayed a mean particle size of 339 ± 14 nm, an entrapment efficiency of 91.07 ± 0.33%, and a Zeta potential of + 26.9 ± 1.2 ± 1.2 mV. In vitro cytotoxicity studies performed on Hela cells indicated a significant (*P* < 0.05) decrease in IC_50_ value around 3-fold compared with pure FBX. The cellular uptake showed a 2-fold increase compared to the free drug. Upon studying the cell death cycle, it was revealed that apoptotic cell death was caused by the drug in the G1/S phase. Altogether, these findings revealed that the optimized chitosomal dispersion exhibited superior cytotoxic activity compared to free FBX, indicating it as a promising efficient and biocompatible delivery system for cervical cancer treatment.

##  Introduction

Cervical cancer is considered the fourth cause of death from cancer in women and comes in the second place as the most prevalent cancer among gynecologic cancers after breast cancer^1^. The traditional applied treatments are surgery, radiotherapy, or radio-chemotherapy. As well as being expensive, these treatments harmfully impact the quality of women’s lives by producing several side effects, like hair loss, immunosuppression, nausea, and frequently have a life-threatening prognosis^2^. Febuxostat (FBX) is an FDA-recognized medication for gout with a proven safety record designed to lower uric acid levels. FBX exists in numerous pharmaceutical dosage forms including emulsions, solid dispersion, and tablets for treatment of hyperuricaemia^3–5^. Recently, it was reported that FBX may act as a protective agent against tumor lysis syndrome in patients with malignant tumors treated by chemotherapy^6^. Moreover, FBX showed cytotoxic actions in various tumor cell lines as A549 non-small cell lung cancer^7^, and HCT116 human colorectal carcinoma inducing apoptotic effects mediated by caspase 3^8^.

Though, many cytotoxic agents when administered in their free form are impeded by poor water solubility, limited bioavailability, rapid metabolic degradation and non-specific distribution, all of which significantly decrease their therapeutic efficacy and raise the risk of adverse effects^9,10^.

Nanotechnology has emerged as a transformative platform in cancer diagnosis and treatment, donating advanced strategies to improve drug solubility, targeting, and therapeutic efficacy. As highlighted in pervious review nanoparticle-based systems can enhance the pharmacokinetic profile of chemotherapeutic agents, enable controlled drug release, and improve tumor accumulation through both passive and active targeting mechanisms. Within this broad class of cancer nanotherapeutics, vesicular systems such as niosomes and liposomes have gained considerable attention due to their biocompatibility and ability to encapsulate hydrophobic drugs. Surface modification of these carriers further refines their performance. In this context, coating with chitosan represents a promising strategy to enhance mucoadhesion, cellular interaction, and localized drug delivery^11^.

Niosomes were found as a carrier system that can encapsulate hydrophilic/lipophilic drugs in a vesicle-like structure and deliver them to their target site in a controlled and/or sustained manner. The structure of niosomes is similar to that of liposomes since it is composed of non-ionic surfactants (such as sorbitan or polysorbates) and cholesterol as a stabilizer. Niosomal features are highly influenced by the type and molar ratio of surfactants and cholesterol, which can affect membrane rigidity, curvature, and vesicle stability. This amphiphilic bilayer structure increases drug bioavailability with low solubility in water by trapping the drug inside the structure and enabling penetration of biological membranes, thus improving its therapeutic effectiveness^12^.

Niosomes are looked as better than other lipid nano-carriers due to the high drug entrapment, slow drug release, improved cellular uptake, biodegradable and biocompatible besides their minimal economic concerns^13^. In addition, their surface can be modified to obtain properties like optimal surface charge or specific targeting^14^. Nowadays, usually decoration of niosomes surface with a mucoadhesive moiety is trending to increase its bioadhesion^15^.

Chitosan (CS) is the most abundant employed cationic polysaccharide prepared by alkali chitin N-deacetylation. CS has special aspects like biocompatibility, non-toxicity, biodegradability, and bioadhesive properties that extensively employed in clinical and biomedical disciplines^16^. Moreover, it exhibits several biological activities, including antioxidant, antimicrobial activity against Gram-positive and Gram-negative bacteria, wound healing capacity, and the in vitro and in vivo ability to complex genetic material. Recently, there has been a huge interest for CS applications in areas such as hematology, immunology, wound healing, drug delivery, food packaging and cosmetics^17^.

Although a number of physiological difficulties may restrict therapeutic efficacy, the vaginal route is a feasible method for targeted cervical drug administration. The acidic pH of the vaginal environment, which ranges from roughly 3.8 to 4.5, constant mucus turnover, enzyme activity, and self-cleaning processes, can all reduce drug retention and cervix penetration. Moreover, the cervicovaginal mucus layer functions as a protective barrier that is semipermeable and may hinder the diffusion and absorption of drugs^18^. Therefore, in order to preserve vesicular stability and achieve regulated drug release, the recommended formulation must be compatible with vaginal pH and vaginal fluids.

There are two different systems to maximize mucosal drug delivery PEGylated niosomes/liposomes and chitosan-coated vesicular systems (chitosomes). To enhance mucoadhesion, prolong residence time, and facilitate cellular absorption by adsorptive endocytosis, chitosomes rely on the cationic nature of chitosan to foster strong electrostatic interactions with negatively charged mucin. This frequently results in increased permeability across mucosal tissues and better local drug retention^19^.

PEGylated vesicles with polyethylene glycol, on the other hand, improve colloidal stability and systemic circulation time by reducing protein adsorption and opsonization through the creation of a hydrophilic steric barrier. However, PEGylation may decrease cellular absorption because of steric hindrance (also known as the “PEG shielding effect”), and its ability to penetrate mucus depends on creating a dense, neutrally charged PEG corona^20^.

In this study, we intended to formulate CS coated niosomes (chitosomes) and assess their applicability as Nano carriers for repurposing the use of FBX delivery for cervical cancer. To prove the possibility of our approach, we performed a detailed physicochemical characterization for the developed FBX-loaded chitosomes, and then evaluate the cytotoxic effect on cervical cancerous cell line (Hela cells).

##  Materials and methods

### Materials

#### Chemicals

Febuxostat was kindly donated as a gift by Eva Company for Pharmaceutical industries, Cairo, Egypt. Sorbitan mono-stearate (Span 60) and Tween 20 were purchased from Oxford Laboratory (Mumbai, India); cholesterol (CHOL) was purchased from Alpha Chemika (Mumbai, India). CS (molecular weight: 150 kDa, deacetylation degree: 75%−85%) was provided from Sigma Aldrich (St. Louis, MO, USA). Porcine gastric mucin (PGM) was purchased from Adamas-beta^®^ (Shanghai, China). HPLC grade methanol, chloroform, acetic acid, and sodium acetate were purchased from Sigma-Aldrich (Steinheim, Germany). Sodium lauryl sulfate (SLS) and hydrochloric acid 30% were purchased from Al-Gomhoria Company for medicines and medical supplies (Cairo, Egypt).

#### Cell line and staining kits

Hela cells were provided by Sigma Aldrich (St. Louis, MO, USA). Cells were stored in RPMI media containing streptomycin (100 mg/mL), penicillin (100 units/mL) and fetal bovine serum (10% v/v) in humidified 5% (v/v) CO2 atmosphere at 37º C. MTT kit purchased from Sigma-Aldrich, Inc. Annexin V-FITC obtained from Bio-Vision Research Products (Linda Vista Avenue, Mountain View, USA). ab139418 kit (Phosphate Buffered Saline (PBS) 100 mL, Propidium iodide (1 mg/mL) 2 mL, and RNaseA (110,000U/mL) 200 µL) provided by (Abcam^®^, UK).

### Experimental design

A full 23 factorial design was constructed to statistically study the effect of formulation variables on the preparation of FBX-loaded chitosomal dispersions using Design-Expert® version 13.0.5.0 software (Stat-Ease, Inc., Minneapolis, Minnesota, USA). As presented in Table 1, the study design involved three formulation factors at two different levels. These formulation factors were the Lipid to drug weight ratio (L/D) (X1) at (7.5 and 15) with respect to FBX concentration (2 mg/ml), the Span 60: Cholesterol: Tween 20 (S60: CHOL: T20) molar ratio (X2) at (1:1:1 and 2:1:1), and CS Concentration (X3) at (0.125 and 0.25% w/v). The studied factors were assessed for their effects on the designated responses; the FBX entrapment efficiency % (EE%; Y1), Particle size (Y2), Zeta potential (Y3). The factors significance in the best-fitting factorial model was evaluated by ANOVA statistical analysis, with statistical significance set at P < 0.05. Response surface plots effectively visualize the relationship between independent variables and one or more response variables.

**Table 1:** The Independent variables and dependent responses of the 2^3^ full factorial design used for development of FBX loaded Chitosomal dispersions.

Independent variables	Levels	
	Low (-1)	High(+ 1)
X_1_: L/D weight ratio	7.5	15
X_2_: S60: CHOL: T20 molar ratio	1:1:1	2:1:1
X_3_:CS %w/v	0.125	0.25
Responses Goals		
(Y1) Entrapment Efficiency (EE)% Maximize (Y2) Particle size (PZ) Minimize (Y3) Zeta Potential (ZP) In range		

### Preparation of FBX-loaded niosomes

Eight FBX loaded niosome formulations were prepared by dissolving 20 mg of FBX and the calculated amounts of S60 and CHOL in 15 ml chloroform: methanol mixture (2:1v/v) in 250 ml rounded bottom flask. Then, the solution was evaporated in a rotary evaporator (IKA, HBIO basic, RV10B S99, Deutschland, Germany) under reduced pressure. The desired amount According to the developed design as shown in Table 2 of T20 was incorporated in hydration medium (10 ml distilled water pre-warmed at 58–60 ^o^C).Under normal pressure at temperature just above the glass transition temperature (Tg) of S60 (58^o^C) the hydration medium was added to dried thin film at 120 rpm for 1 h until a milky white dispersion was obtained confirming niosomes formation.

### Preparation of FBX-loaded chitosomes

Niosomal dispersions were coated with (CS) at concentrations of 0.125%, and 0.25% w/v. Low molecular weight CS was dissolved in acetic acid solution (0.1% v/v), sonicated then filtered. The coating process was achieved by adding an equal volume of different concentrations of CS solutions on niosomal dispersion and stirring (100 rpm) for 2 h at room temperature^21^. The developed dispersion systems were sonicated for 5 min at room temperature to achieve particle size uniformity. Moreover, blank Chitosomal dispersions were prepared with identical procedure in absence of FBX and stored with the FBX-chitosomal dispersions at 4 °C till further investigation.

###  Characterization of chitosomal preparation

#### Determination of entrapment efficiency % (EE %)

The entrapment efficiency percent in each formula was calculated in triplicate using direct method. 1 ml of each formula and also blank was centrifuged using a cooling centrifuge at 4^o^C and 20,000 rpm for 1 h. The supernatant was removed and the chitosomal pellets were washed with distilled water. The pellets were re-dispersed, re-centrifuged and the collected pellets were dissolved and vortexed with 3 ml of pure chloroform for 1 min in order to break up the chitosomal particles. Then, further dilution of dispersion with 10 ml methanol to dissolve all the entrapped FBX. The same steps were applied on the blank chitosomal pellets, and the concentration of FBX in methanolic solution was determined at λ_max_ (314 nm) using a UV/visible spectroscopy (Jasco spectrophotometer, Japan). The EE % of FBX into chitosomal formulae was calculated from this Eq. (1)

1 $$\:EE\%=\frac{\mathrm{A}\mathrm{m}\mathrm{o}\mathrm{u}\mathrm{n}\mathrm{t}\:\mathrm{o}\mathrm{f}\:\mathrm{e}\mathrm{n}\mathrm{t}\mathrm{r}\mathrm{a}\mathrm{p}\mathrm{p}\mathrm{e}\mathrm{d}\:\mathrm{F}\mathrm{B}\mathrm{X}}{\mathrm{O}\mathrm{r}\mathrm{i}\mathrm{g}\mathrm{i}\mathrm{n}\mathrm{a}\mathrm{l}\:\mathrm{a}\mathrm{m}\mathrm{o}\mathrm{u}\mathrm{n}\mathrm{t}\:\mathrm{o}\mathrm{f}\:\mathrm{F}\mathrm{B}\mathrm{X}\:\mathrm{a}\mathrm{d}\mathrm{d}\mathrm{e}\mathrm{d}}x\:100$$

####  Determination of zeta potential, particle size and polydispersity index

The zeta potential, particle size and polydispersity index (PDI) of eight prepared chitosomal dispersions were determined using dynamic light scattering integrated in Malvern Zetasizer (Malvern instruments/Worcestershire, UK). Chitosomal samples were diluted with suitable volume of de-ionized water before measurement to avoid multi-scattering phenomena. Measurements were performed in triplicate for each sample at 25 °C and results were represented as mean value ± standard deviation.

#### Optimized formulation prediction

The optimum FBX-loaded CHITOs (FBX- CHITOs _OPT_) preparation was estimated based on data statistical analysis and various response optimizations taken from the Design-Expert program. The predicted formulation was prepared and characterized for its particle size, efficiency of entrapment, and Zeta potential. Moreover, the FBX- CHITOs _OPT_ formulation was subjected to the further subsequent examinations.

#### Differential scanning calorimetry (DSC)

DSC thermograms of pure FBX, S60, CHOL, T20, CS, and the quinary physical mixture of FBX with all ingredient at ratio of (1:1:1:1:1 w/w), and FBX- CHITOs OPT were examined using DSC-50, Shimadzu; Kyoto, Japan. Samples (4 mg) were fixed in standard aluminum pan and heated at a constant heating rate of 10 °C / min to a temperature of 400 °C under a flow of nitrogen gas with flow rate 25 ml/min to avoid sample oxidation^22^.

#### Fourier transform infrared (FTIR)

FTIR was performed to study any chemical interaction between FBX and other components of the optimized formula. The IR spectra of the samples mentioned previously in DSC section were recorded using IR Spectrophotometer (Shimadzu 8400 S, Lab Wrench, Japan). The samples were finely grinded with 100 mg of dry potassium bromide powder, compressed into transparent disc and scanned over the range 400 to 4000 cm-1^22^.

#### Transmission electron microscopy (TEM)

The morphological examination of FBX- CHITOs _OPT_ dispersion was examined using transmission electron microscopy (TEM). The freshly prepared chitosomal system was diluted with deionized water, and one drop was placed on a carbon coated copper mesh and left to dry. The sample was then visualized directly without staining using transmission electron microscope (JEOLJEM-1400, USA). And photographs were taken at suitable magnifications^23,24^.

#### In-vitro drug release from FBX chitosomal dispersions

For FBX- CHITOs _OPT_ formulation, Chitosomal pellet equivalent to 10 mg FBX were separated by cooling centrifugation at 4o C and 20,000 rpm for 1 h and washed with distilled water. Then, the chitosomal pellet was re-suspended in distilled water. The in-vitro release of FBX from the obtained Chitosomal dispersion was performed using dialysis bag membrane. The dialysis bag containing chitosomal dispersion was placed in 50 ml of 0.1 M acetate buffer (pH 4.5) containing 1% SLS (mimicking vaginal pH) to maintain sink condition for 30 h^25^. The release medium was stirred magnetically at 100 rpm and the temperature was maintained at 37 ± 0.5C.

At various time intervals, 3 ml samples were withdrawn and replaced with 3 ml of fresh medium to maintain sink conditions. The samples were measured spectrophotometerically at λ_max_ (314 nm). The release data were calculated in triplicate and then mean values were plotted as percentage cumulative drug release against time. The drug release study was compared with the same amount of pure FBX to investigate the variance in release pattern^26^.

#### Release kinetics and mechanisms^27^

DD Solver^®^ software is used to statistically assess release data to fit the release profiles to various kinetic models such as zero-order, first-order, Higuchi, and Korsmeyer- Peppas to detect the model with the highest regression coefficient (R^2^), and exponent “n” in the Korsmeyer–Peppas model expect the release mechanism^28^.

#### In-vitro bioadhesion studies

Mucoadhesive strength of the produced chitosomal dispersion can be measured qualitatively by (measuring the change in zeta potential) and quantitative (measuring the adsorption of mucin on the formulations). Porcine gastric mucin (PGM) powder was dispersed in acetate buffer solution (PH 4.5) overnight to prepare mucin powder dispersion of 0.1% w/v. An equal volume of FBX loaded chitosome suspension mixed with Mucin dispersion and kept at 37 ◦C for 48 h under gentle stirring. Zeta potentials of the prepared mixture and the mucin dispersion were determined using Malvern Zetasizer (Malvern instruments/Worcestershire, UK)^29^. Stock solution of mucin was diluted to different concentrations 25, 50, 75, 100, 125 and 150 µg/mL. The absorbance of each dilution was measured by UV spectrophotometer at λ_max_ 258 nm. The calibration curve was plotted and the linear regression equation and R^2^ were then determined (Y = 0.0058x + 0.0203; R^2^ = 0.9981). Furthermore, the prepared mixture was centrifuged at 11,000 rpm for 1 h, and the supernatant was separated. The free mucin concentration was analyzed by a UV spectrophotometer (Jasco spectrophotometer, Japan) at 258 nm^21^. The bioadhesive strength of PGM with CHITOs _OPT_ was calculated using the Following Eq. (2):

2 $$\:"Mucin\:binding\:\%="\:\:"Ci-Cf"\:/"Ci"\:\:"x\:100\:"\:$$

Where *Ci* is the initial mucin amount used and *Cf* is the free mucin amount in the supernatant.

### Stability studies

The stability of the chitosomal dispersion was evaluated at refrigerated and room temperature 4 and 25 °C, respectively for three months. Physical stability was monitored, and the effects of temperature and time on the PS, ZP, EE%^30^.

###  In vitro cell culture studies

####  In vitro assessment of cytotoxic potential by MTT Assay

The Cell viability study was performed on cervical cancerous cells (Hela cells) and The IC_50_% values of Hela cells treated with Blank CHITOs, pure FBX, or FBX- CHITOs _OPT_ for 24 h were measured by the MTT assay^31^. Briefly, the cell suspension (3 × 10^5^ cells) was seeded into a 96-well tissue culture plate and incubated in a humidified environment (5% CO_2_, 37 °C) to allow the complete cell attachment. The cells were incubated for 48 h with 100 µL of different concentrations (0.4, 1.6, 6.3, 25, and 100 µg/mL) of FBX solution, FBX- CHITOs _OPT_, and blank CHITOs. MTT solution was added to each well at 10% of the culture medium volume. Following a further 4 h of incubation, MTT solubilization solution was put in a volume equal to the original culture medium volume to dissolve the resulting formazan crystals. Spectrophotometerically measure absorbance at a wavelength of 570 nm. The IC_50_ for Blank CHITOs, free FBX, and FBX- CHITOs _OPT_ was calculated based on the curves obtained measuring the variation of cell viability (%) as a function of increasing concentrations.

#### Cellular uptake of drug in cervical cancerous cell line (Hela cells)

The internalization of the optimized formulation was studied in vitro using Hela cell cultures. At a density of 5000 cells per well and 100 µL volume of medium, the cells were seeded in 96-well plates and allowed to adhere for 24 h before the experiment. Three differentiating cell line groups (untreated, treated with a concentration equivalent to IC50 of free FBX and /or FBX- CHITOs _OPT_) were incubated for 12 h. Then, the collected growth media were washed with Phosphate-Buffered Saline (PBS) three times. FBX concentrations in the collected media and PBS washes were analyzed using HPLC/MS^32^. The % cumulative intracellular FBX was calculated based on the following Eq. (3):

3 $$Cumulative{\text{ }}\mathrm{int} racellular{\text{ }}FBX{\text{ }} = {\text{ }}\left[ {\left( {C_{0} \_{\text{ }}C_{{exa{\text{ }}t}} } \right)/C_{0} } \right]{\text{ }}X{\text{ }}10$$

Where C_0_ is the initial concentration of FBX added to cells and C _exa t_ is the extracellular concentration of FBX at time = t.

#### Cell cycle analysis by flow cytometry

Propidium iodide flow cytometry is intended for quantitative DNA content analysis in tissue culture cells. After 24 h of incubation of Hela cells (3 × 10^5^ cells per well) at 37 °C in a 5% CO_2_ atmosphere, the culture medium was substituted with freshly prepared medium including either free FBX or FBX- CHITOs _OPT_ at IC_50_ concentrations, then the cultured cells were incubated for another 24 h. The cell pellets were separated then re-suspended in a mixture of PBS and fixed in 70% ethanol for 2 h at 4 °C. Another cycle of centrifugation was performed, and rehydrated the cells with PBS. Then, the produced cellular pellets were kept within a mixture of propidium iodide and ribonuclease for 30 min before investigation. Data were presented as % relative to the untreated cell population^33^.

#### Annexin V staining

The quantification of apoptotic cells was performed using a dual staining reported method (annexin V fluorescein isothiocyanate (V-FITC) and propidium iodide)^34^. Hela cells were seeded at a density of 4 × 10^5^ cells per well in six-well plates overnight. Then, fresh medium containing either free FBX or FBX- CHITOs _OPT_ was added over the seeded cells for 24 h. Subsequently, the cells were collected by centrifugation (1000 rpm, 5 minutes), re-suspended in a buffer solution, and stained with V-FITC and PI according to the kit’s protocol. The stained cells were incubated at 25 °C for 5 minutes in the dark, and apoptotic cells were counted using flow cytometry.

## Results and discussion

### Factorial design data analysis

A 2^3^ complete factorial designed study was applied to examine the impacts of three independent factors: Lipid to drug ratio (X_1_), S60: CHOL: T 20 molar ratio (X_2_), and CS Concentration (X_3_) at two levels on EE % (Y_1_), particle size (Y_2_), and Zeta potential (Y_3_). The results for each response were analyzed using Design Experts software; ANOVA was used to assess the influence of the independent variables on the responses, considering *P* < 0.05 for statistical significance. The software identified the best fitting model (linear, 2F1, and/or quadratic) for each response depending on the predicted and adjusted R^2^ values. The 2F1 model was the best-fitting model prospecting for all responses (% EE, PS, and ZP) since the adjusted R^2^ with a responsible agreement with predicted R^2^ (the difference is less than 0.2) and adequate precision was greater than 4.

#### Effects of experimental factors on FBX entrapment efficiency % (EE %) (Y1)

**Fig. 1:**
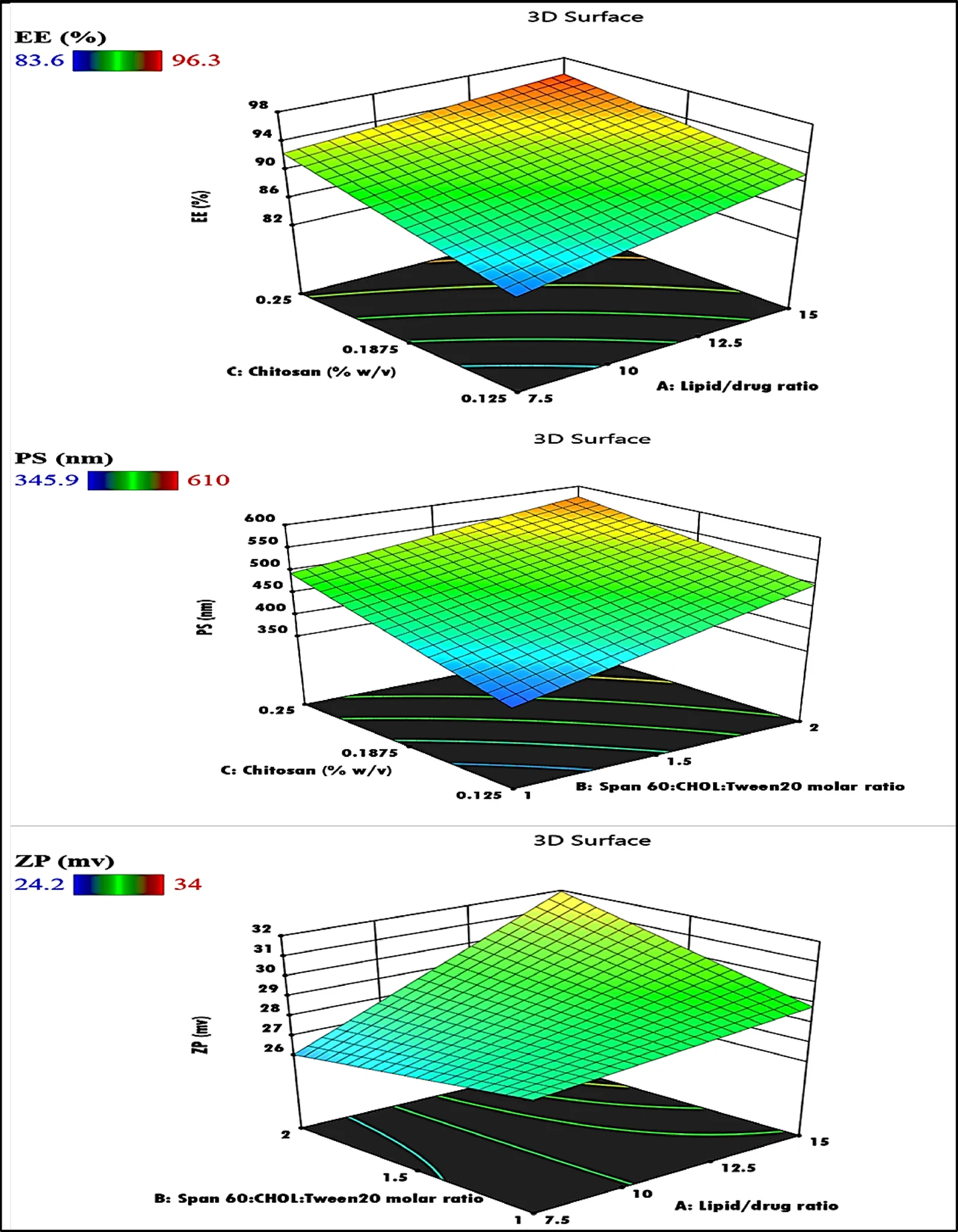
the 3D plots of the two-factor interaction effect of independent parameters on (A) Entrapment Efficiency %, (B) Particle size, and (C) Zeta potential.

The EE% of FBX within Chitosomal formulae ranged from 83.6 ± 1.3% to 96.3 ± 1.7% as summarized in Table 2. Statistical analysis of the 2FI model using ANOVA revealed that the model terms (X1 = Lipid/drug weigh ratio, X2 = S60: CHOL: T20 molar ratio and X3 = CS Concentration (%w/ v)) as well as the two factor interaction (X1 × 3) had significant impact (*P* < 0.05) on EE %( Y1) while the two factor interactions (X1 × 2 and X2 × 3) were insignificant (P˃ 0.05). These effects are distinctively observed in 3D response surface graphs Fig. 1A. The polynomial Eq. (4) relating the effect of formulation variables on the EE% (Y1) in terms of coded values was:

4 $$\begin{aligned} Y1 & = 91.0313 + 2.40625*X1 + 0.91875*X2 + 2.96875*X3 \\ & - 0.15625*X1X2 - 0.75625*X1X3 - 0.16875*X2X3 \\ \end{aligned}$$

Accordingly, from the coefficients of model terms, the individual effect of (X1) was significantly positive on EE%. Increasing Lipid to drug ratio from 7.5 to 15 was found to increase EE%. Febuxostat is a drug that is poorly soluble in water and has a high affinity for lipid environments, classifying it as a BCS class II compound. As a result, increasing the lipid content creates a larger hydrophobic matrix for accommodating the drug, which enhances the partitioning of FBX into the lipid bilayer and improves the entrapment efficiency^35^. Similar outcomes have been observed for lipophilic drugs that are incorporated into vesicular lipid-based systems^36^. Also, increasing the concentration of lipid bilayer ingredients (S60, T20 and CHOL concentration) will certainly increase multilamellar and/or multivesicular vesicles with larger accessible space for drug entrapment which means higher loading capacity^37^.

Concerning S60: CHOL: T20molar ratio (X2), it was observed that (X2) showed a significant (*P* < 0.05) positive effect on EE%. This might be due to many suggestions. Firstly, S60 molecules have long alkyl chain and low hydrophilic-lipophilic balance (HLB) that could improve FBX EE% by increasing its hydrophobic molecules solubility and permeability into niosomes vesicles^38^. Secondly, EE% decreased with increase in concentration of T20 (decrease X2) and increased with increase in the concentration of S60 and maintained lower level of T20 (increased X2) because the combination of both HLB values of T20 (16.7) and S60 (4.7) were taken together, which further confirmed increased entrapment efficiency and maintained hydrophilic-lipophilic balance^39^. Finally, increasing CHOL levels (decreased X2) was previously reported increasing EE% due to the increased hydrophobicity and stability of the vesicles^40,41^.

However, in our study the effect of increasing S60 levels was more noticeable. Also, CHOL could compete with drug molecules for the packing space within the bilayer, preventing the drug from being entrapped during the hydration process^42^. Furthermore, CS Concentration (%w/ v) (X3) affected EE% positively. This may be ascribed to CS formed surface coating over the lipid bilayer of niosomes and prevented the leakage of the drug^43^.

####  Effect of experimental factors on Particle Size (Y2)

Vesicular size determination is considered one of the most important aspects, particularly for vesicles intended for vaginal drug delivery. The average particle size (in nanometer) of the eight formulated FBX Chitosomal dispersions ranged from 345.9 ± 8 to 610 ± 17 nm as summarized in Table 2. Generally, the size affects the vesicular ability to release and retained the drug inside the mucosal layer. The particle size between 200 and 500 nm is considered as ideal to enhance drug retention inside vaginal mucosa; instead, the surface area increased in the case of small vesicles, can determine a rapid release of the drug and consequently its quick leakage from the vaginal cavity^44^.

statistical analysis of the 2FI model using ANOVA revealed that the model terms (X1 = Lipid/drug weigh ratio, X2 = S60: CHOL: T20molar ratio and X3 = CS Concentration (%w/ v))as well as the two factor interaction (X2 × 3) had significant impact (*P* < 0.05) on PS (Y2) while the two factor interactions (X1 × 2 and X1 × 3) were insignificant (P˃ 0.05). These effects are distinctively observed in 3D response surface graphs (Fig. 1B). The polynomial Eq. (5) relating the effect of formulation variables on the PS (Y2) in terms of coded values was:

5 $$\begin{aligned} Y2 & = 482.05 + 23.625*X1 + 54.925*{\text{ }}X2 + 52.425*X3 \\ & + 8.4*X1X2 + 1.4*X1X3 - 13.9*X2X3 \\ \end{aligned}$$

Regarding the effect of lipid/drug ratio (X1) on the vesicle size (Y2), it was evident that increasing niosomal components total amount was accompanied with a mutual increase in niosomal vesicle size at constant level of S60: CHOL: T20 (X2). This effect might be ascribed to the existence of a considerable amount of film forming components relative to the hydration medium. This might trigger the formation of multilamellar and/or multivesicular vesicles upon hydration, resulting in a considerable increase in niosomal vesicle size^40^.

From the coefficients of model terms, the individual effect of S60: CHOL: T20 molar ratio (X2) was significantly positive on PS. The observed effect of the Span 60/Cholesterol/Tween 20 molar ratio on the vesicular characteristics can be explained based on previously reported studies. Formulations containing higher amounts of Span 60 and cholesterol (e.g., F4 and F6) exhibited larger particle sizes. Since S60 possesses a long saturated alkyl chain (C18), which increases the hydrophobic character of the bilayer and reduces its curvature, resulting in larger particle size^45,46^. Also, Cholesterol played a vital role in stabilizing the bilayer by reducing membrane fluidity and permeability, thus contributing to increased rigidity and particle size at higher concentrations^47^.

On the other hand, increasing Tween 20 content (e.g., F3 and F8) presented higher bilayer flexibility due to its hydrophilic nature and bulky head group, which enhanced membrane curvature and limited excessive vesicle growth^48^. Therefore, the balance between Span 60, Tween 20, and cholesterol determines the final vesicular structure.

Furthermore, the effect of CS concentration % w/v (X3) on niosomal size was marginally increased on increasing the CS concentration. This increase may be attributed to the chemical interactions between the CS hydrogen bond and surfactant head groups. Accordingly, a coating layer was a rounded on the surface of the niosome^49^.

####  Effect of experimental factors on Zeta potential (Y2)

The zeta potential of FBX within Chitosomal formulae ranged from 25.1 ± 1.3 to 34 ± 1.7 mV as summarized in Table 2. statistical analysis of the 2FI model using ANOVA revealed that the model terms (X1 = Lipid/drug weight ratio and X3 = CS Concentration (%w/ v))as well as the two factor interactions (X1 × 2) had significant impact (*P* < 0.05) on Zeta potential (Y3) while (X2 = S60: CHOL: T20 molar ratio) and the two factor interactions (X1 × 3 and X2 × 3) were insignificant (P˃ 0.05). These effects are distinctively observed in 3D response surface graphs (Fig. 1C). The polynomial Eq. (6) relating the effect of formulation variables on the Zeta potential (Y3) in terms of coded values was:

6 $$\begin{aligned} Y3{\text{ }} & = 28.575 + 1.8*X1 + {\text{ }}0.4*X2 + 2.2*X3 \\ & + 1.175*{\text{ }}X1X2 + 0.075*X1X3 - 0.275*X2X3 \\ \end{aligned}$$

From the coefficients of model terms, the individual effect of (X1) was significantly positive on zeta potential. Increasing Lipid/drug ratio from 7.5 to 15 was found to increase zeta potential. It was previously reported that increasing CHOL concentration introduces a negative charge onto the vesicular surface due to the uneven polarity distribution of CHOL hydroxyl group^35,46^, As well as, increased surfactant concentration that cause an increase in the electrical conductivity^50^. The positive charge reflects chemical interactions between the CS hydrogen bond and surfactant head groups. So, increasing the concentration of bilayer-forming ingredients will definitely increase the number of the formulated niosomes and subsequently more vesicles accessible to be coated. The individual effect of (X3) was significantly positive on zeta potential. Increasing concentration of CS from 0.125 to 0.25%w/v (X3) also leads to increasing Zeta potential (Y3) due to increasing of cationic-charged CS molecules. The presence of CS on the surface of the niosome indicates high stability due to electrostatic interaction between niosomes and Chitosan^43^.

**Table 2:** Entrapment efficiency%, mean zeta potential, particle size, polydispersity index, and desirability of the prepared chitosomal formulae (FBX amount 20 mg).

Formula	S60	CHOL	T20	CS	EE% (Y1)	PS(Y2)	ZP(Y3)	Desirability
F1	32	28	90	12.5	83.6 ± 0.25	345.9 ± 8	25.1 ± 0.7	0.066
F2	52	23.4	74.6	25	93.2 ± 0.31	541 ± 11	27.8 ± 0.82	0.442
F3	63	57	180	25	95 ± 0.15	509 ± 2.6	31.3 ± 1.15	0.584
F4	104.3	46.7	149	25	96.3 ± 0.11	610 ± 17	34 ± 3.9	0.063
F5	52	23.4	74.6	12.5	86.2 ± 0.12	468.9 ± 4	24.2 ± 2.7	0
F6	104.3	46.7	149	12.5	92.1 ± 0.08	528 ± 16	29.9 ± 4.6	0.454
F7	32	28	90	25	91.5 ± 1.2	477.9 ± 22	30 ± 0.71	0.588
F8	63	57	180	12.5	90.35 ± 2.04	375.7 ± 35	26.3 ± 0.5	0.685

###  Optimization of FBX-loaded chitosomes formulation

**Fig. 2:**
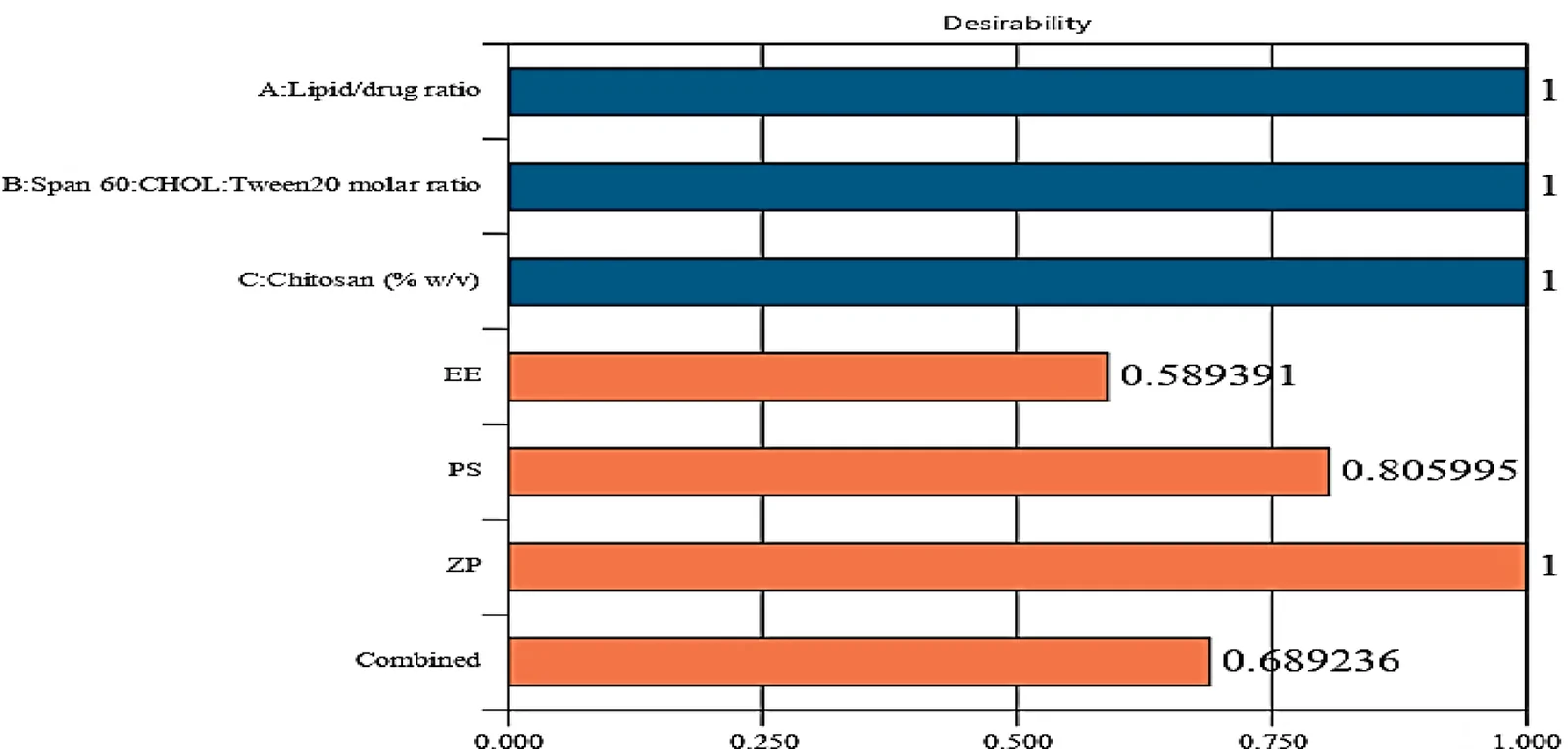
The bar graph of desirability of F9.

Afterward 2^3^ factorial analyses for eight formulations to study the effects and interaction of formulation parameters on responses, an optimization step was performed using Design Expert^®^ software. According to the constraints provided previously in Table 1, the desirability for each formulation was obtained by software as showed in Table 2. The recommended formulation F9 with a composition of Lipid/ drug ratio, S60: CHOL: T20 molar ratio, CS concentration % w/v had the least PS and highest % EE with a reasonable ZP compared to other formulations and showed higher desirability value of 0.689 as illustrated in Fig. 2. F9 was prepared and the actual results of EE%, PS, and ZP were presented in Table 3 and Compared with the predicted values. There was statistically non- significant difference between predicted and actual values. As shown in Table 3, the % deviation didn’t exceed 1. The percentage deviation for each response was determined and was calculated from Eq. (7)

7 $$\% deviation = \frac{{\left| {\Pr edicted~value - ~Experimental~value} \right|}}{{\Pr edicted~value}} \times 100$$

As shown in Table 3, the % deviation didn’t exceed 1.

**Table 3:** The composition and Validation of the optimized FBX-loaded Chitosomal dispersion (F9).

F9 suggested composition			
Parameters	X1 = L/D weight ratio	X2 = S60: CHOL: T20 molar ratio	X3 = CS (%w/ v)
	15	1:1:1	0.147

Responses	Predicted value	Experimental value	% Error
%EE	91.14	91.07	0.08%
Particle size	399.7	399	0.17%
Zeta potential	27.16	26.9	0.95%

### Differential scanning calorimetry (DSC)

**Fig. 3:**
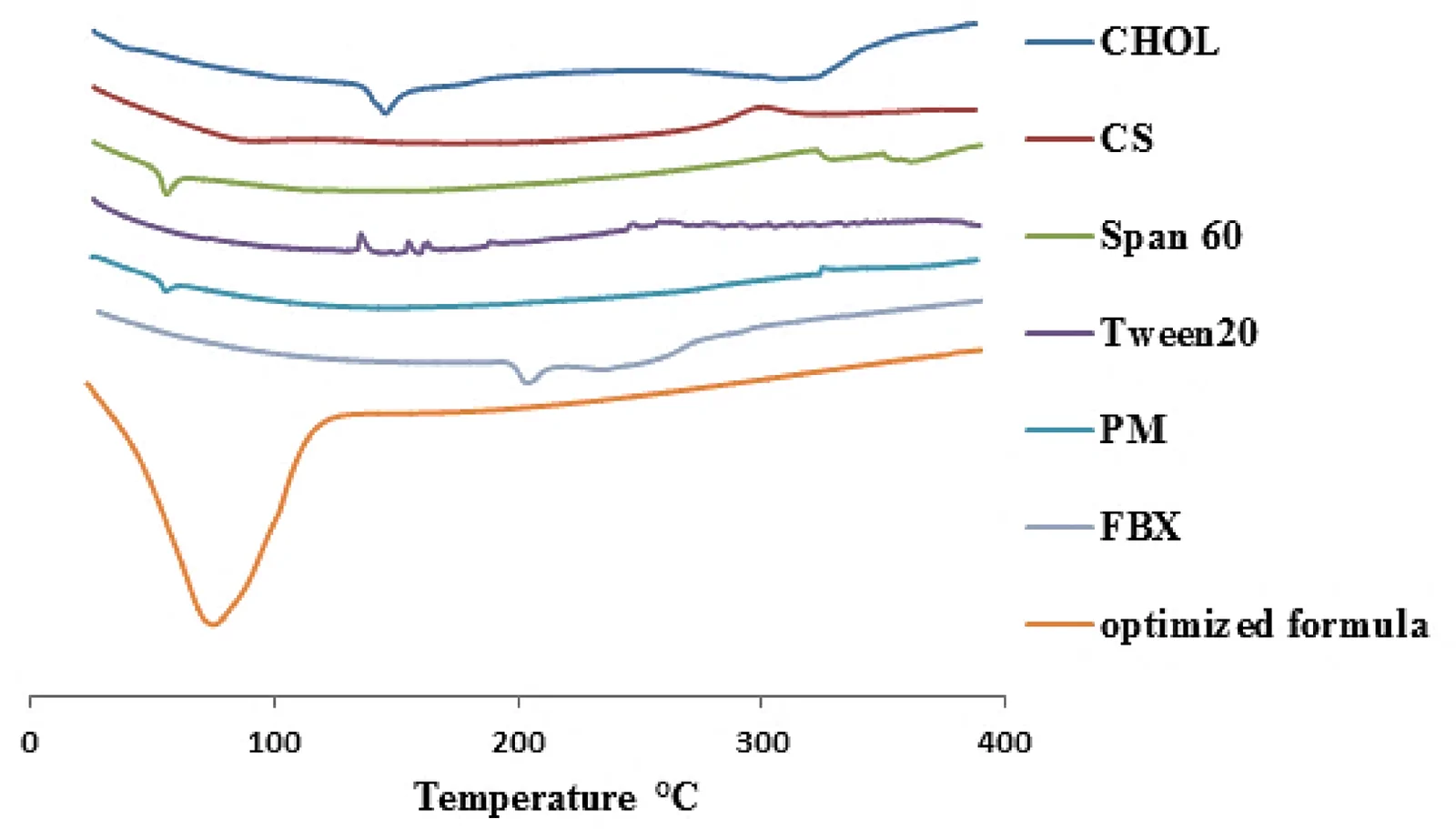
Overlay of DSC thermograms of FBX, the different Chitosomal forming excipients, and F9.

Figure 3 showed the DSC thermograms of FBX, S60, CHOL, T20, CS, physical mixture and FBX- CHITOs OPT (F9). FBX was characterized by a sharp endothermic peak at 209.3 °C, corresponding to its melting point^27^. S60 had a narrow and sharp endothermic peak at 56.95 °C corresponding to its glass transition temperature (Tg) while CHOL showed a broad endothermic melting transition started at 40 °C most likely due to the loss of water molecules, and a sharp endothermic melting peak at 145.45 °C followed by rapid degradation of CHOL^51^. T20 was exhibited exothermic peaks at 130 °C with two exothermic peaks are due to undergo thermal oxidation and weight loss under oxidative atmosphere^52^. Moreover, CS showed an endothermic peak at 73.63 °C reflecting water loss; besides, an exothermic peak is evident at 289.02 °C reflecting CS degradation^24^. Physical mixture thermogram revealed only the melting endotherm of S60 without the presence of peaks corresponding to other components, due to the dilution effect^53^. DSC thermogram of F9 showing no characteristic peak of FBX, only the CS peak was observed at the low melting region. The absence of FBX endothermic peaks suggests drug incorporation into chitosomes^54^.

###  Fourier transform infrared (FTIR)

**Fig. 4:**
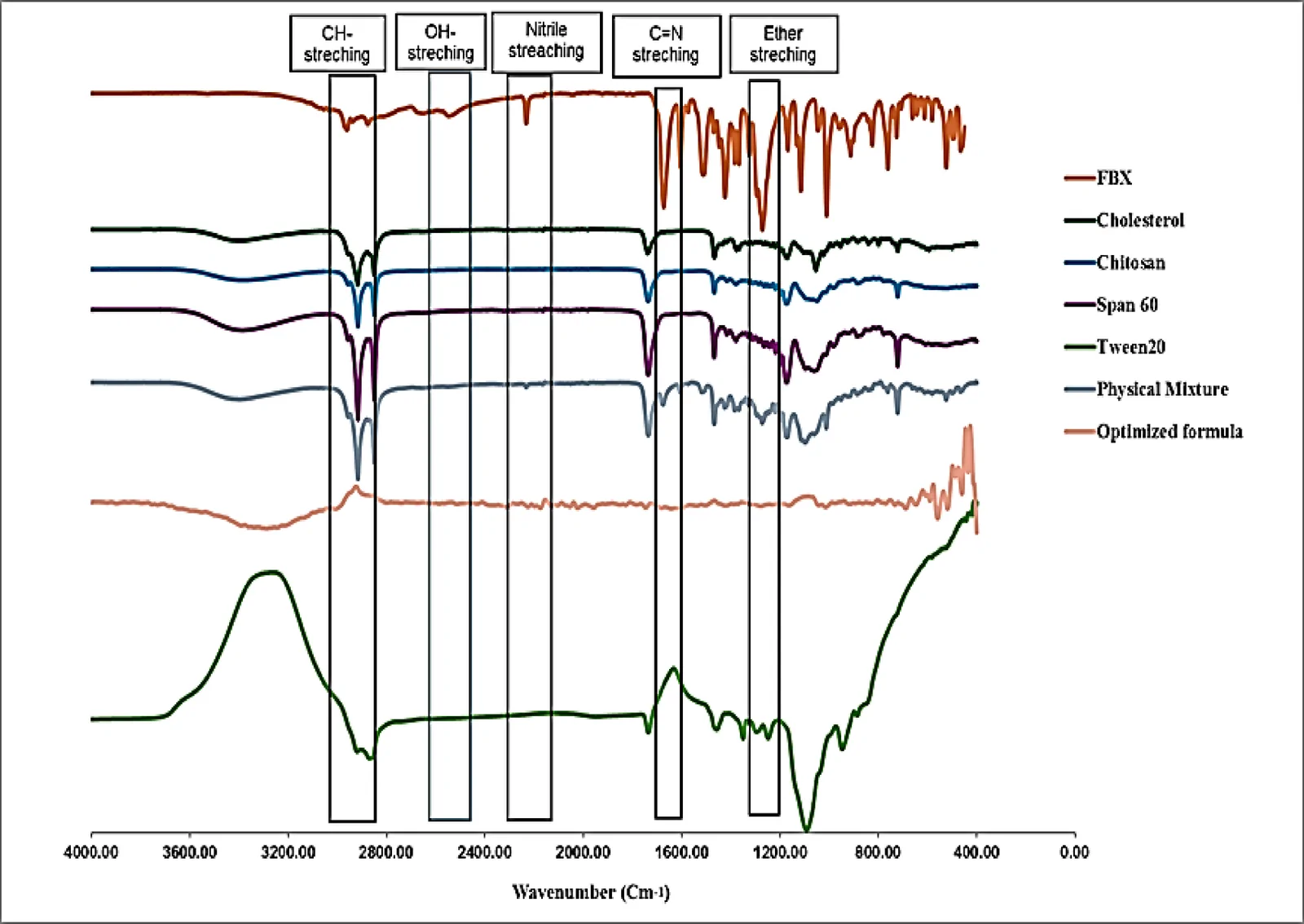
Overlay of FTIR spectra of FBX, the different Chitosomal forming excipients, and F9.

The FTIR spectra of FBX, S60, CHOL, T20, CS, Physical Mixture, and F9 are presented in Fig. 4. The main absorption bands of FBX were 2962.66 cm − 1 and 2873.93 cm − 1 (alkane - C H group), 2546.04 cm − 1 (hydroxyl group), 2233.87 cm − 1 (C ≡ N nitrile stretch), 1681.93 cm − 1 (C = N stretching of thiazole ring) and 1276.88 cm − 1 (ether group)^26^. S60 showed specific bands of hydroxyl groups (-OH), alkyl groups (-CH), and esters (R-CO-OR’) at 3425, 2918, and 1738 cm-1, respectively. While, CHOL had absorption bands of hydroxy groups (-OH) 3447 cm-1, aromatic carbon (CH-CH) 2931 cm-1, and carboxylate group (R-CO-OH) 1704 cm-1^12^. T20 showed five characteristic bands at 1249, 1467, 1736, 2871, and 2923 cm-1 which are associated with the stretching of ester group C–O, bending of CH2 group, stretching of ester C = O group, and symmetric and asymmetric stretching of alkyl C–H bonds respectively and a characteristic band at 3300–3400 cm-1 belongs to the stretching of–OH bond^55^. CS showed a characteristic vibrational peak at 3420.74 cm − 1 of NH stretching, 3123.98 cm − 1 of OH stretching, 1559.63 cm − 1 of NH bending, and 1101.84 cm − 1 of C–O stretching assuring the chemical structure of CS^26^. The IR spectrum of physical mixture showed characteristic peaks belonged to FBX along with S60, CHOL, T20, and CS, indicating that there is no interaction between FBX and chitosomal constituents. Also, IR spectrum of Optimized formula showed characteristic peaks of FBX didn’t appeared may have been due to solubilization of drug in lipid layer^39^. The intense peaks appeared at 3298.75 cm − 1 (OH stretching), 2925 cm − 1 (C-H peaks) 1637.51 cm − 1 (NH bending), 1085 cm − 1 (C–O stretching) which related to CS coat. Slight changes in the characteristic peaks were observed for optimized formula due to formation of vesicles^56^.

### Transmission electron microscopy

**Fig. 5:**
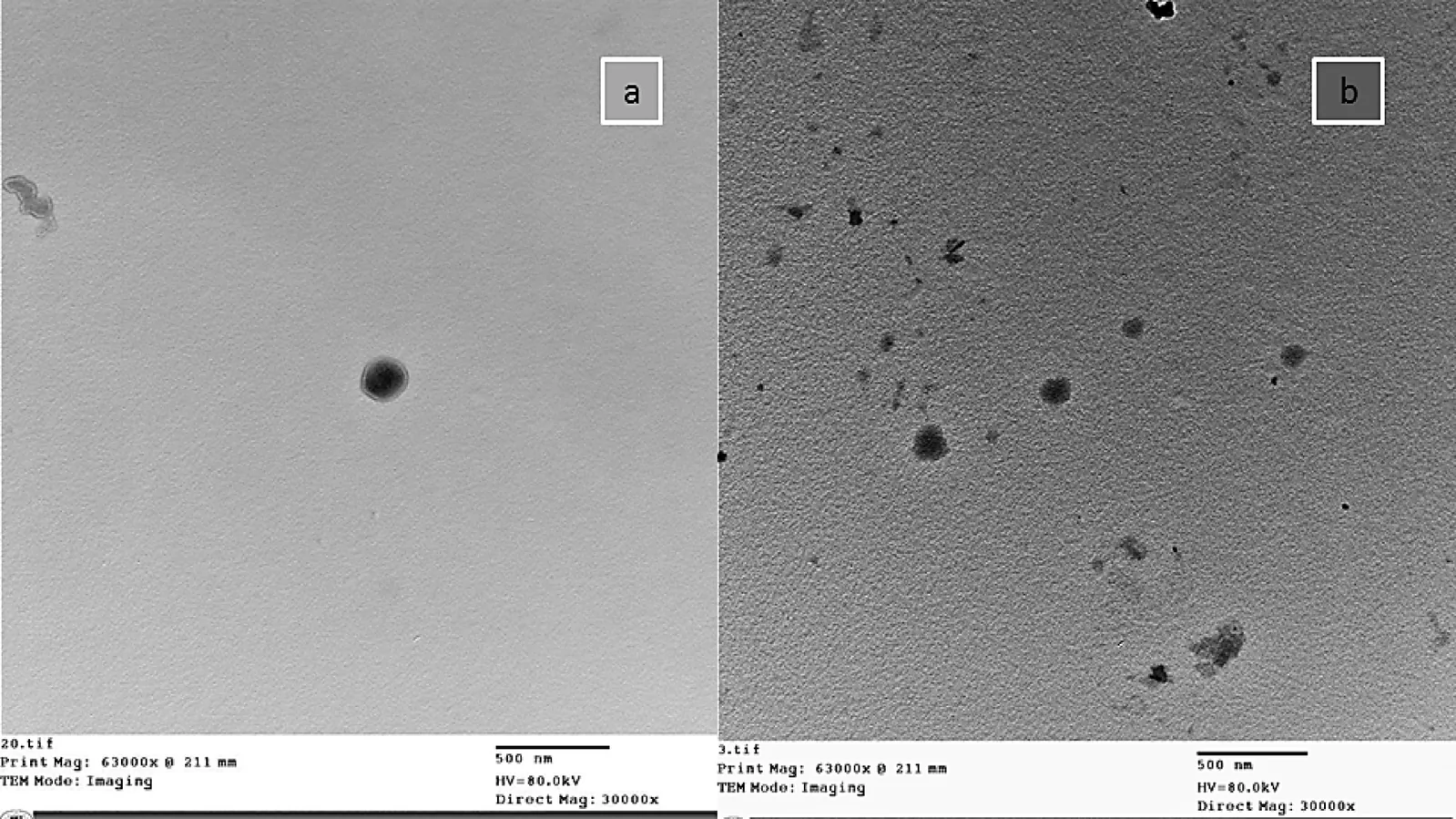
Transmission electron photomicrographs of niosomes: (**a**) FBX loaded niosomes coated with chitosan(F9),and (**b**) un coated niosome.

FBX loaded niosomes with and without coatings were prepared, and TEM was used to visualize the morphology of those vesicles Fig. 5. TEM images revealed that both of the coated and uncoated niosomes have distinct circular shape. The uncoated niosomes showed intense vesicles with definite margins, distributed homogeneously. Meanwhile, F9 showed an inner core, surrounded by a relatively faded cover and double layer boundaries. These results are confirming the successful coating of FBX niosomes and formulation of chitosomes^41^.

### In vitro release profile of free and encapsulated FBX

**Fig. 6:**
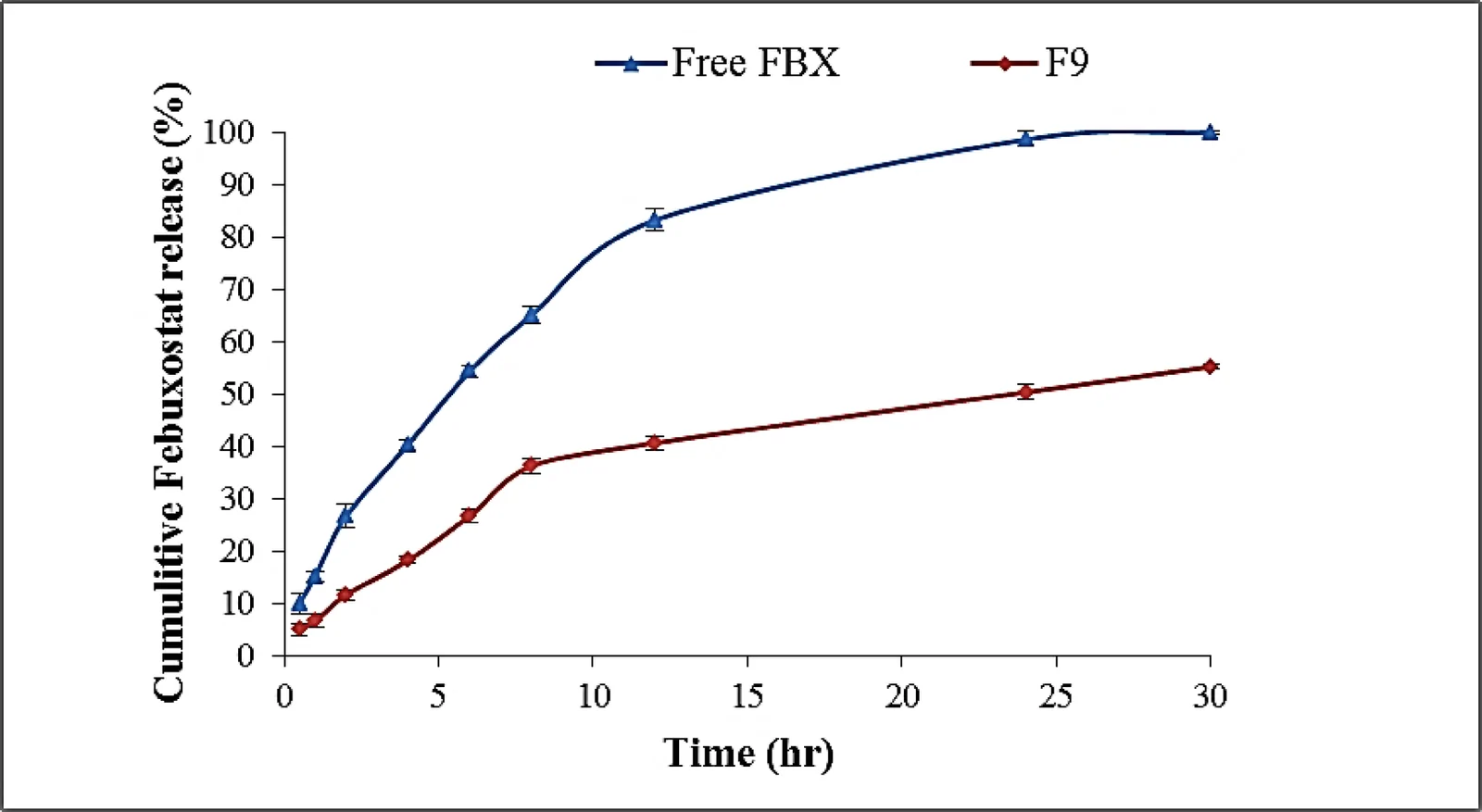
% Cumulative drug release versus sampling time of free drug and FBX- CHITOs _OPT_ (F9) in acetate buffer (pH = 4.5) containing 1% SLS.

Figure 6 shows the cumulative release profiles of FBX before and after encapsulation into the prepared chitosome over a period of 30 h. The cumulative release of F9 was considered as the control release when compared with the free FBX release profile in conditions similar to vaginal environment. The cumulative release profiles of F9 showed that the amount of FBX released after 6 h was approximately around 25% in acetate buffer (1% SLS) pH 4.5, while free drug release profile showed faster release of FBX 54.3 ± 1.4%. After 30 h F9 displayed sustained release effect when compared with free FBX solution. The cumulative drug released from FBX-loaded CHITOs (F9) was 55.2 ± 0.55%, while free FBX solution showed 100% of drug released^40^. Predictably, the FBX release from chitosome was to be prolonged due to CS coating, which provides an external physical barrier enclosing the niosomes^57^.

### Release kinetics

The release measurement was analyzed using different release kinetic models to reveal the FBX release mechanisms from chitosomal dispersion. The resulting correlation coefficients (r^2^) were showed that FBX release was in agreement with Korsmeyer-Peppas (R^2^ =0.9761), providing a prolonged drug release system involving numerous processes, including diffusion, swelling and erosion. The n values used for explication of drug release mechanism from the prepared chitosomal dispersions were determined from the slope of the plot of log cumulative percent of drug release (≤ 60%) versus log time. Where, *n* ≤ 0.45 indicates a Fickian diffusion mechanism. Values of (0.45 < *n* < 0.89) corresponds to non-Fickian anomalous transport while *n* = 0.89 corresponds to Case II (polymer relaxational transport). If *n* > 0.89, this indicates super case II transport^28^. The obtained n value from Korsmeyer-Peppas was 0.5, indicating non-Fickian anomalous transport. Similar kinetic results have been investigated with CS coated formulations in the literature^24,58^.

### In-vitro bioadhesion studies

**Fig. 7:**
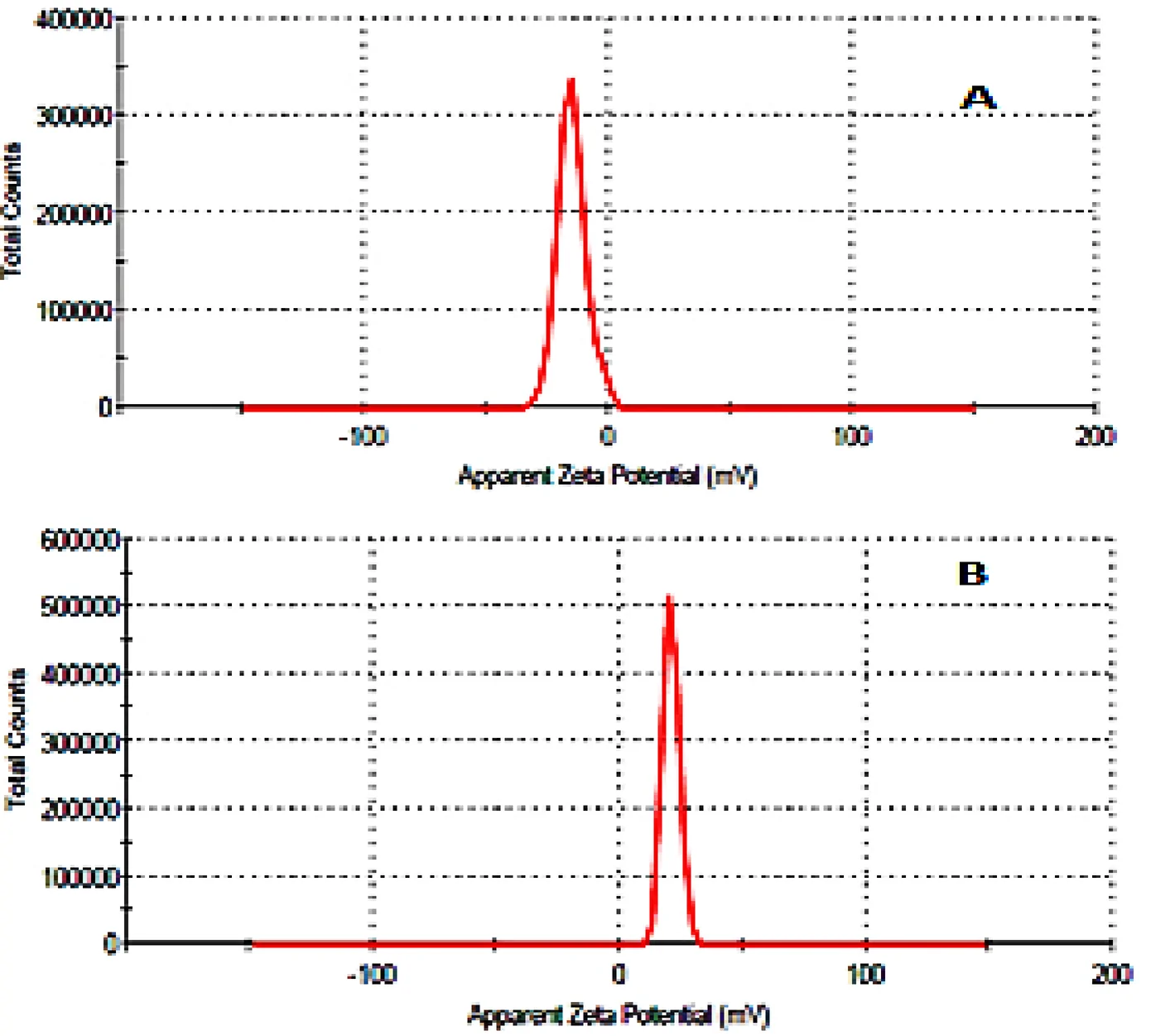
Zeta potential of pure mucin dispersion (**A**) and mucin-chitosome mixture (**B**).

One of the most studied activities for chitosomes is the mucoadhesive property. The zeta potential of the pure mucin and mucin-chitosome mixture was determined to be -10.04 ± 0.53 and + 20.1 ± 0.15, respectively, as shown in Fig. 7. The mucin binding % of FBX-loaded CHITOs was 64.32 ± 0.23%. These finding attributed to the interaction between the amine group (NH3 +) of chitosan existing on chitosome surface and the carboxylate (COO -) or sulfonate (SO3̵) group of mucin^59^. Furthermore, the enhanced mucoadhesion will provide a longer residence time and help achieve enhanced permeation and therapeutic efficacy^60^.

### Stability studies

A stability study is essential to the assessment of any formulation. It is crucial to analyze the entrapment efficiency, particle size, and zeta potential during the storage period to determine stability of vesicles. The stability of the vesicles during the storage period is indicated by the plateau of these measurements, which supports drug retention. Table 4 shows the results of the stability study of the optimized chitosomal dispersion, which was conducted for three months at both room temperature (25 °C) and refrigerated temperature (4°C). The ANOVA results displayed insignificant (*p* < 0.05) changes in EE%, PS, and ZP at the refrigerated temperature compared to the initial data. The physical instability issues of the particles are generally reduced when vesicular systems are stored at a refrigerated temperature (4 °C)^61^.

In contrast, PS and ZP were significantly (*p* < 0.05) changed during storage at room temperature; meanwhile, EE% was insignificant (*p* < 0.05) relative to the results at zero time. Increasing of particle size at room temperature could be related to the affinity of the particles to fuse and aggregate^62^. Notably, the optimized chitosomal formulation showed higher stability at refrigerated temperature than that at room temperature^62^.

**Table 4:** Stability study of the optimized chitosomal dispersion at refrigerated temperature (4 °C ± 1 °C) and at room temperature (25 °C ± 2 °C).

Storage period	Evaluation Parameter					
	Refrigerated Temperature (4 °C ± 1 °C)			Room Temperature (25 °C ± 2 °C)		
	EE%	PS(nm)	ZP(mv)	EE%	PS(nm)	PS(nm)
Initial	91.07 ± 0.05	399 ± 6	26.9 ± 0.7	91.07 ± 0.05	399 ± 6	26.9 ± 0.8
Month1	91.33 ± 0.23	440 ± 8	26.2 ± 0.4	93.05 ± 0.26	549 ± 22	27.8 ± 0.6
Month2	91.01 ± 0.21	412 ± 12	25.8 ± 0.5	92.61 ± 0.12	718 ± 15	29.2 ± 0.4
Month3	91.04 ± 0.33	397 ± 10	26.4 ± 0.5	94.32 ± 0.34	664 ± 9	31.6 ± 0.3

### In vitro cell culture studies

#### Cytotoxic effect/s of FBX and FBX- CHITOs _OPT_

**Fig. 8:**
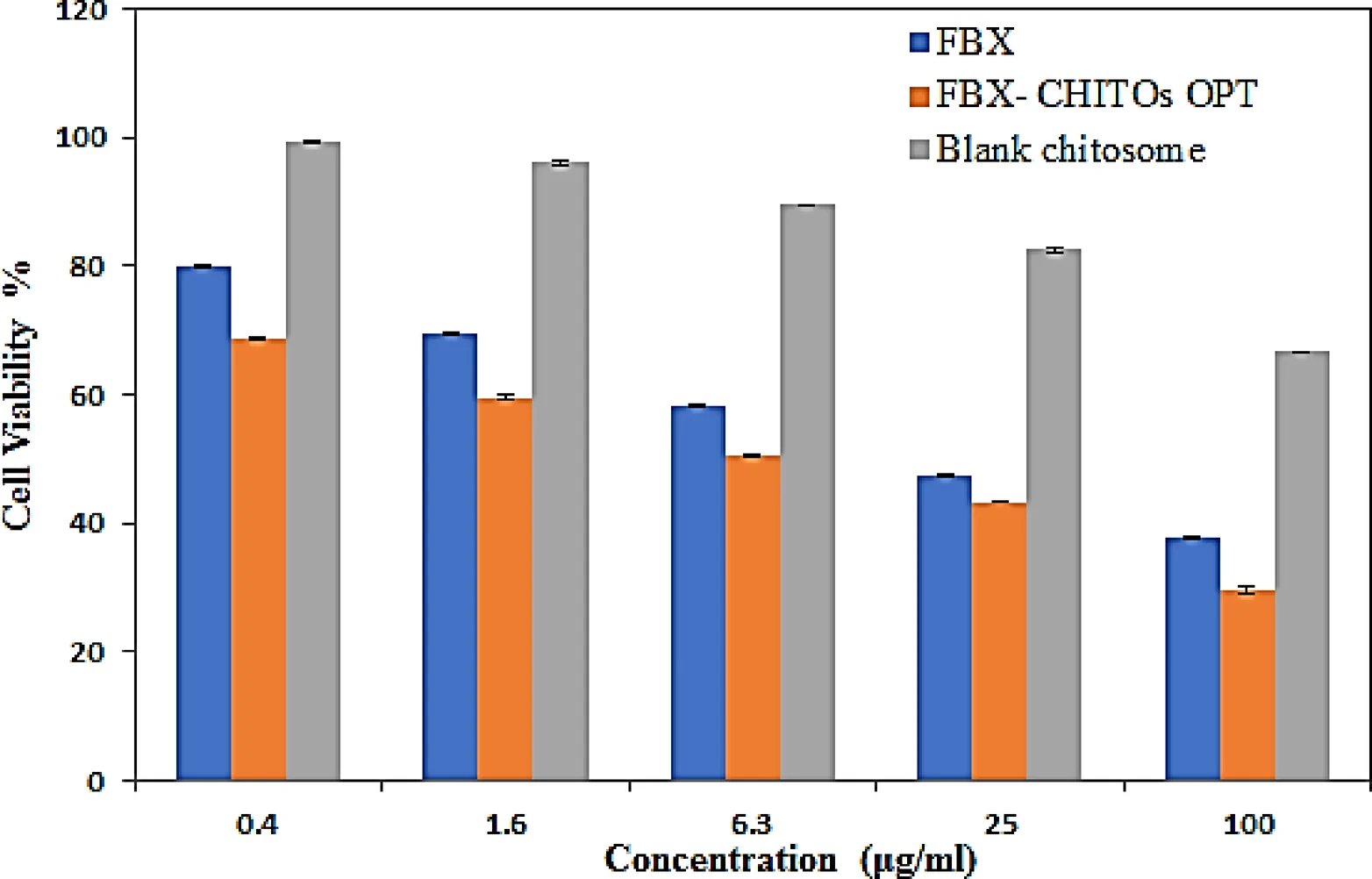
Impact of Blank chitosome, pure FBX, and optimized FBX-CHITOs treatments on Hela cell viability. Data are expressed as mean ± SD (*n* = 3).

Evaluation of cytotoxic potential of a drug against cancerous cells has been the most extensively used assay^63^. Therefore, the effect of FBX and its nano-formulation (FBX-loaded CHITOs) was evaluated on cervical cancer cell line (Hela cells)^64^. After 24 h of treatment, both FBX as well as F9 showed a dose-dependent response, evident by the cell viability in Fig. 8. Overall, the cytotoxicity of F9 was more noticeable (nearly three folds) and significant (*p* ≤ 0.05) compared to FBX alone, and blank chitosome suggestive of a better cytotoxic potential of Febuxostat when used as a nano-formulation. All the results of IC_50_ values were statistically significant (*P* < 0.05) and indicated that blank chitosomes exhibited minimal toxicity for tested cell line, confirming the safety and biocompatibility of that carrier. The IC_50_ values of F9, FBX, and blank chitosome were (6.615 ± 0.2 µg/mL), (19.36 ± 0.58 µg/ mL), and (3991 ± 120 µg/mL), respectively. The higher cytotoxic effect of FBX-loaded CHITOs may be due to the smaller size of the nano-formulation, thereby enabling its passive transport into the cell compared to free FBX. The results in agreement with previous formulated FBX nano-formulation tested for their cytotoxicity^7,8^.

#### Cellular uptake and drug accumulation

Quantitative analysis of intracellular FBX amount further confirmed the advantage of CS coating. After incubation for 12 h, intracellular FBX concentration of F9 and free FBX were 318 ± 11.07 ng/ml, and 161 ± 8.29 ng/ml, respectively. The cellular uptake was 2 fold increases in accordance with the prolonged adhesion on the surface of cellular monolayers of positively charged chitosome than free drug. Subsequently, the best cellular growth inhibition was achieved with FBX-loaded CHITOs. The strongest anti-proliferative effect of FBX-loaded CHITOs was consistent with its highest intracellular drug content and better in vivo pharmacological antitumor activity^65^.

#### Cell cycle analysis

**Fig. 9:**
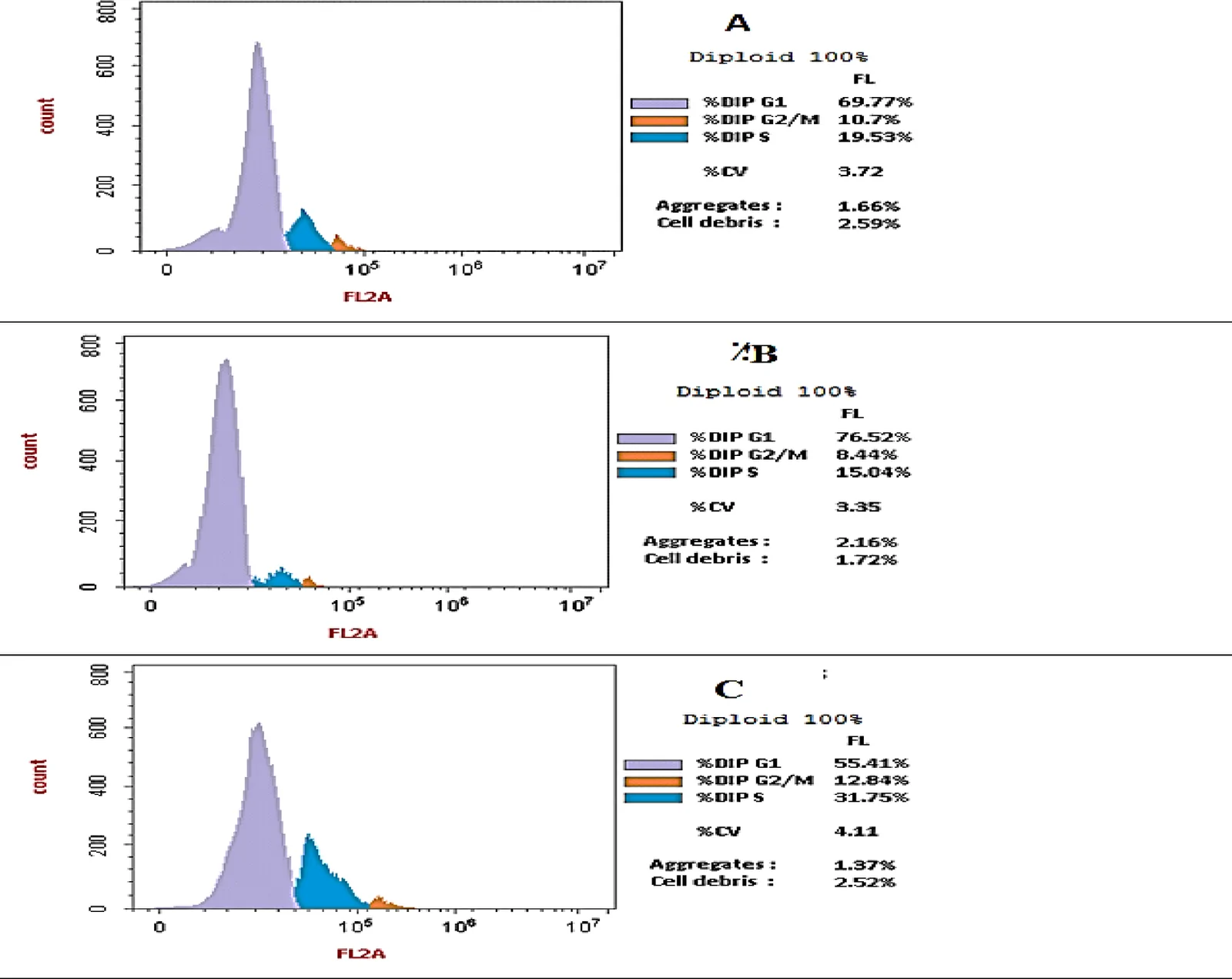
Flow cytometry cell cycle quantitative analysis of free FBX solution (**A**), FBX-loaded CHITOs (F9) (**B**), and control (**C**).

Flow cytometry analysis was used to assess the effect of FBX-loaded CHITOs on the cell cycle progression of cervical cancer cells (Hela cells) compared with free FBX solution. It was found that both FBX and F9 treated groups significantly induced cell cycle arrest compared to control group. The results of untreated cells % values for G0/G1, S, and G2-M equal to 55.41, 31.75, and 12.84 respectively as showed in Fig. 9. As illustrated in Table 5, F9 significantly inhibited the proliferation of Hela cells by inducing G1/S phase arrest. FBX-loaded CHITOs showed an increase in the proportion of cells in the G0/G1 phase, exceeding the effect observed with free FBX. This G0/G1 phase cell cycle arrest was followed by decreasing in the % of cells in the S and G2/M phases following treatment with FBX-loaded CHITOs (Tables 5 and 6). These outcomes indicate that chitosomal nanoparticle treatment impaired the cell cycle progression of cervical cancer cells, leading to accumulation in the G0/G1 phase.

**Table 5:** the effect of free FBX and FBX-loaded CHITOs (F9) on the redistribution of growth-arrested Hela cells in the different phases of the cell cycle.

Sample	DNA content		
	%G1	%S	%G2/M
Free FBX	69.77	19.53	10.7
F9	76.52	15.04	8.44
Control cells	55.41	31.75	12.84

#### Effect of FBX and FBX-loaded CHITOs on apoptosis induction

**Fig. 10:**
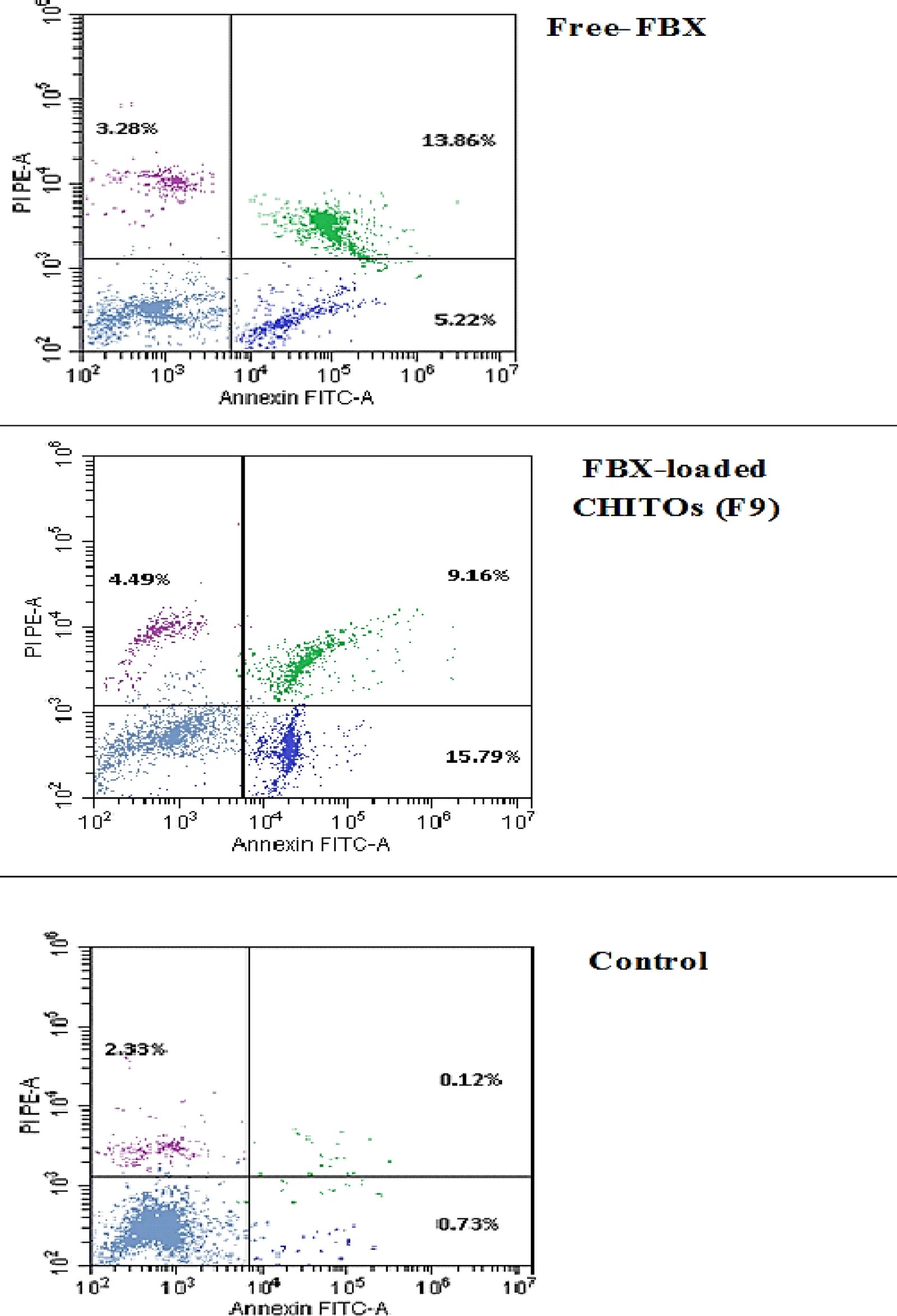
Cellular apoptosis of cervical cancer cells evaluated via flow cytometry using annexin V-FITC staining.

To study the underlying mechanism of cytotoxicity induced by FBX and F9, effect on cell apoptosis was studied by employing Flow cytometry, using annexin V/FITC kit. It was observed that FBX (19.36 ± 0.58 µg/ mL/24 h.) significantly induced cellular apoptosis compared to the control; however, treatment with F9 (6.615 ± 0.2 µg/mL /24 h.) significantly increased the proportion of apoptotic cells compared to either control or FBX-treated Hela cells. As illustrated in Fig. 10 the top left quadrant (Q1) represents cells undergoing necrosis, the top right quadrant (Q2) depicts cells in late apoptosis and necrosis, the bottom left quadrant (Q3) indicates healthy cells, and the bottom right quadrant (Q4) shows cells in early apoptosis. Observing the results, FBX-loaded CHITOs treated groups induced significantly cell necrosis (4.49%) compared to FBX (3.28%) alone or control (2.33%) groups. Additionally, the percentage of sum total of early and late apoptotic cells in F9 (29.44 ± 2.57%) treatment group was significantly higher compared to either FBX (22.36 ± 0.37%) or control (3.18 ± 0.09%), suggestive of a strong pro-apoptotic potential of FBX chitosomal nano-formulation. Earlier reports in different biological models have shown that Febuxostat reduces reactive oxygen species (ROS) generation through xanthine oxidase inhibition, thereby influencing mitochondrial dependent apoptotic pathways, including modulation of Bcl-2/Bax expression, cytochrome c release, and caspase activation^66^. In addition, Febuxostat has been reported to regulate key signaling pathways such as MAPK and NF-κB, which are known to be involved in apoptosis and cell survival^67^. Thus, regulation of these oxidative stress and mitochondria dependent apoptotic processes may be responsible for cytotoxic and apoptotic effects of FBX in cervical cancer cells in current investigation.

**Table 6:** Summary of the percentage of cell apoptosis.

Sample	Apoptosis%			
	Total	Early	Late	Necrosis
Free FBX	22.36	5.22	13.86	3.28
F9	29.44	15.79	9.16	4.49
Control cells	3.18	0.73	0.12	2.33

## Conclusion

This study successfully developed and evaluated FBX-Chitosomes as an innovative drug delivery system aimed at enhancing anticancer efficacy against cervical cancerous cells (Hela cells). The formulated FBX-loaded chitosomes were characterized for effective vesicles formation. In-vitro cytotoxicity studies demonstrated superior effects for FBX chitosomes than free FBX. FBX Chitosomes offer a promising and effective nanocarrier for cervical cancer therapy. Future studies should emphasis on comprehensive in vivo evaluation, including pharmacokinetic analysis and therapeutic assessment in relevant cervical cancer animal models, to validate the translational potential of FBX loaded chitosomal system.

## Data Availability

The datasets generated during and/or analysed during the current study are available from the corresponding author on reasonable request.
